# Systematic review of published trials: long-term safety of topical corticosteroids and topical calcineurin inhibitors in pediatric patients with atopic dermatitis

**DOI:** 10.1186/s12887-016-0607-9

**Published:** 2016-06-07

**Authors:** Elaine C. Siegfried, Jennifer C. Jaworski, Jennifer D. Kaiser, Adelaide A. Hebert

**Affiliations:** Saint Louis University, Cardinal Glennon Children’s Hospital, 1465 South Grand Avenue, St Louis, MO 63104 USA; Prescott Medical Communications Group, 205 North Michigan Avenue, Suite 3400, Chicago, IL 60601 USA; University of Texas-Houston Medical School, 6655 Travis, Suite 980, Houston, TX 77030 USA

**Keywords:** Atopic dermatitis, Long-term safety, Lymphoma, Pediatric, Pimecrolimus, Tacrolimus, TCS, TCI, Topical calcineurin inhibitor, Topical corticosteroid

## Abstract

**Background:**

Many clinicians have concerns about the safety of atopic dermatitis (AD) treatments, particularly in children requiring long-term daily maintenance therapy. Topical corticosteroids (TCS) have been widely used for >5 decades. Long-term TCS monotherapy has been associated with adverse cutaneous effects including atrophy, rebound flares, and increased percutaneous absorption with potential for adverse systemic effects. Topical calcineurin inhibitors (TCIs), tacrolimus and pimecrolimus, available for 1–2 decades, are not associated with atrophy or increased percutaneous absorption after prolonged use and have much lower potential for systemic effects. However, since 2006 TCIs have carried a controversial Boxed Warning based on a theoretical risk of malignancy (eg, skin and lymphoma) that has limited TCI use for standard-of-care maintenance therapy.

**Methods:**

A comparative systematic search of PubMed was done for long-term (≥12 week) clinical trials of TCS or TCI treatment in patients <12 years with AD. Citations were reviewed for inclusion based on MeSH terms, abstracts, and relevant article text. Studies were excluded if they did not encompass subjects <12 years, or were <12 weeks’ duration, retrospective, meta-analyses, or limited to anecdotal case reports.

**Results:**

Of 27 trials meeting criteria, 21 included 5825 pediatric patients treated with TCIs, and 6 included 1999 patients treated with TCS. TCS studies were limited to low- to mid-potency products, and all but one study lacked a vehicle control. Eight TCI studies were vehicle-controlled, and safety data were well reported, with ≤5 % of patients reporting discontinuation due to adverse effects (DAEs). Cutaneous and systemic adverse events (AEs) were similar in TCI and vehicle groups, with no reports of lymphoma. Safety data in TCS trials were less well reported. DAE incidence was addressed in just 2 trials, and systemic and cutaneous AEs were mostly unreported.

**Conclusions:**

Data supporting long-term use of TCIs are robust, documenting safety and efficacy, while data supporting long-term TCS use are limited to low- to mid-potency products. Our review identifies a lack of information on the safety of commonly prescribed, long-term monotherapy with mid- to high-potency TCS in pediatric AD, and supports standard-of-care maintenance therapy with TCIs and intermittent use of low- to mid-potency TCS for flares.

## Background

Atopic Dermatitis (AD) is a chronic, pruritic inflammatory skin disease that occurs most frequently in children. It is the most common chronic pediatric inflammatory skin disease, affecting 12.5 % of US children (aged 0–17 years) from 2009 to 2011, an increase of 5.1 % from 1997 to 1999 [1]. More than half of pediatric patients with AD have mild disease [2], yet the majority of pediatricians refer even their mild patients to dermatologists after providing initial, limited care [3, 4]. This pattern of referrals is due at least in part to questions about the safety of using topical corticosteroids (TCS) and topical calcineurin inhibitors (TCI) to treat AD, particularly in pediatric patients. However, given the shortage of specialists and an increased emphasis on the accountable care model, primary care physicians and pediatricians will continue to play an important role in the management of AD, both as a first-line contact and in regular maintenance following consultation with a specialist.

TCS, which are considered to be first-line treatment for AD flares, have been FDA-approved for a variety of grandfathered indications since 1955. Their mechanism of action, though not well understood, is multifaceted and includes broad-spectrum impact on immune and skin barrier function. Despite their demonstrated efficacy in AD, TCS are associated with a known potential for cutaneous atrophy-related adverse effects such as telangiectasia, striae and purpura, as well as focal hypertrichosis, hypopigmentation and perioral dermatitis [5–9]. In addition, long-term and/or more than once-daily use is associated with subclinical barrier disruption that can result in rebound flares following discontinuation. Long-term subclinical barrier disruption may also cause cumulative increases in percutaneous absorption, with the possibility of rare but insidious and difficult-to-quantify systemic adverse events such as adrenal suppression, poor growth, hypertension, hyperglycemia, insulin resistance, and cataracts [10–14]. These safety concerns are increased in pediatric patients, whose greater body surface area-to-weight ratio is thought to cause increased percutaneous absorption. This risk may be compounded by concomitant use of corticosteroids for other atopic comorbidities (asthma, allergic rhinitis).

In 2000–2001, TCIs were approved in the US for “short-term and noncontinuous chronic treatment of AD in nonimmunocompromised individuals who have failed to respond adequately to other topical prescription AD treatments” [15, 16]. Tacrolimus ointment is available in 2 concentrations: 0.03 %, approved for patients over age 2, and 0.1 %, approved only for patients over age 16 due to theoretical concerns. Pimecrolimus 1 % cream is approved for patients over age 2. In 2006, the FDA instituted a Boxed Warning for both TCIs based on a theoretical risk of malignancy (including lymphomas) that sparked a debate over their safety and appropriate use [17].

Since then, no clear link has been demonstrated between TCI use and lymphoma risk, despite almost a decade of clinical and epidemiological studies, post-marketing surveillance, and monitoring of reports to the FDA Adverse Event Reporting System (AERS). Recent published reviews and/or meta-analyses that assess lymphoma risk of TCIs based on the last decade of clinical experience conclude that there is no evidence that TCI use is associated with increased risk of lymphoma (Table 1) [18–39]. Yet the Boxed Warning remains, leaving many clinicians hesitant to prescribe TCIs despite their potential benefit to some patients. Unlike TCS, TCIs do not carry the risks of skin atrophy, percutaneous absorption, or rebound flares, and have been also been demonstrated to reduce TCS use in long-term studies [40–48]. Therefore TCIs are potentially useful as steroid-sparing agents, and as first-line topical anti-inflammatory treatment on the face and in skin folds.

**Table 1:** Summary statements from review articles that assess TCI lymphoma risk^a^

Citation	Evidence of lymphoma risk with TCIs?		Lymphoma risk summary statement
	Yes	No	
Berger 2006 [18]		*√*	…no causal proof that TCIs cause lymphoma…
Deleuran 2009 [19]		*√*	…no studies support that the use of topical immunosuppression increases the risk of local or systemic cancer^b^.
Ehrchen 2008 [20]		*√*	…no data indicating that topical therapy in humans results in an increased risk for lymphomas.
Fonacier 2005 [21]		*√*	…risk/benefit ratios of topical pimecrolimus and tacrolimus are similar to those of most conventional therapies…
Langley 2007 [22]		√	…there is no clinical evidence to establish that treatment with pimecrolimus cream 1 % increases the risk of malignancy.
Lebwohl 2006 [23]		√	…no causal relationship between the use of TCIs and the occurrence of lymphoma…
Legendre 2015 [24]		√	…systematic literature review shows slightly increased risk of lymphoma in patients with AD…role of topical steroids and TCIs is unlikely to be significant.
McNeill 2007 [25]		*√*	…low incidence of lymphoma and lack of temporal relationship points to a strong safety profile thus far in regards to tacrolimus and lymphoma.
Munzenberger 2007 [26]		√	…no data that show that TCIs are associated with an increased risk of lymphoproliferative disease…lymphoproliferative disease was induced only when doses of TCIs well above the maximum recommended human doses were used.
Orlow 2007 [27]		√	…no evidence to suggest that there is any increased risk of malignancy associated with TCIs.
Ormerod 2005 [28]		√	…no evidence to date to suggest an increased risk of cutaneous or visceral cancer.
Ortiz de Frutos 2008 [29]		√	…with the current information, it cannot be associated to an increase of any type of neoplasms^b^.
Patel 2007 [30]		√	…no established causal link between the topical immunomodulators tacrolimus and pimecrolimus and…malignancy.
Ring 2008 [31]		*√*	…the potential risk of malignancy seems to be low.
Rustin 2007 [32]		√	…no evidence of a causal link between the use of tacrolimus ointment and the rare cases of skin cancer that have been reported.
Sánchez-Pérez 2008 [33]		√	…there doesn’t exist scientific evidence of increase of skin cancer, lymphomas or systemic immunosuppression in patients that use…topical tacrolimus^b^.
Spergel 2006 [34]		√	…studies from clinical trials, systemic absorption, and post-marketing surveillance show no evidence for this systemic immunosuppression or increased risk for any malignancy. …no evidence of increased incidence of lymphoma with short-term or intermittent long-term …tacrolimus and pimecrolimus…
Tennis 2011 [35]		*√*	…the hypothesis that pimecrolimus and tacrolimus cause malignancy…has not been supported by the epidemiological studies to date…
Thaçi 2007 [36]		√	…current scientific data do not support increased concern for risk of malignancy.
Thaçi 2010 [37]		√	…no scientific evidence of an increased risk for malignancy due to a topical treatment with calcineurin inhibitors.
Weischer 2007 [38]		*√*	…tumor risk of topical immunomodulators is lower than the FDA black box warning may indicate.
Werfel 2009 [39]		√	…clinical studies with pimecrolimus have not shown any evidence of an increased risk of malignancy…analysis of spontaneously reported adverse events has also not shown any evidence of malignancy…

^a^Review articles or meta-analyses that assess the lymphoma risk of TCIs were identified by querying PubMed with the terms (lymphoma OR neoplasm OR malignancy OR cancer) AND (topical calcineurin inhibitor OR TCI OR pimecrolimus OR tacrolimus) AND (atopic dermatitis OR eczema), and filtering for meta-analysis, review, and systematic review articles. Articles were excluded if lymphoma risk was not the main focus of the article, or if authors did not come to a conclusion regarding lymphoma risk (or merely referenced conclusions from other papers)

^b^Statement is quoted from the translated abstract of a foreign-language article

To assess the safety of TCS and TCI use in children, we performed a comparative systematic literature search for published, long-term clinical trials of TCS or TCI treatment in pediatric patients with AD.

## Methods

### Systematic searches for published, long-term clinical trials of TCI and TCS trials in pediatric patients

**Fig. 1:**
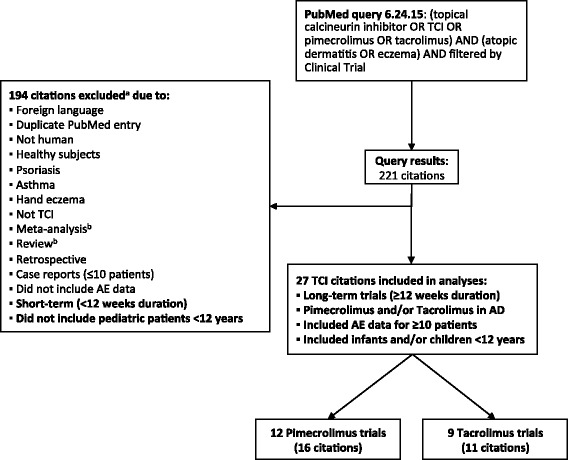
Systematic search strategy for published long-term (≥12 weeks) TCI trials in pediatric patients (<12 years) with AD. ^a^ Exclusions were based on review of MeSH terms, abstract, or (when necessary) the article text. ^b^ Meta-analyses and reviews were excluded if no new (previously unpublished) data were presented

**Fig. 2:**
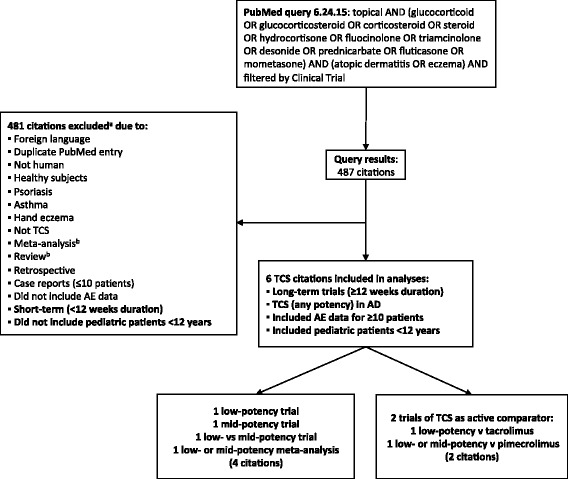
Systematic search strategy for published long-term (≥12 weeks) TCS trials in pediatric patients (<12 years) with AD. ^a^ Exclusions were based on review of MeSH terms, abstract, or (when necessary) the article text. ^b^ Meta-analyses and reviews were excluded if no new (previously unpublished) data were presented

The search strategies we used to identify published, long-term (≥ 12 week) clinical trials of TCI and TCS in pediatric patients (<12 yrs of age) with AD are shown in Figs. 1 and 2. For TCI trials, PubMed was queried with the following terms: (topical calcineurin inhibitor OR TCI OR pimecrolimus OR tacrolimus) AND (atopic dermatitis OR eczema). Results were filtered for clinical trials to eliminate other types of publications (eg, review articles, meta-analyses, case reports) and minimize risk of bias. For TCS trials, PubMed was queried with the terms: topical AND (glucocorticoid OR glucocorticosteroid OR corticosteroid OR steroid OR hydrocortisone OR fluocinolone OR triamcinolone OR desonide OR prednicarbate OR fluticasone OR mometasone) AND (atopic dermatitis OR eczema). Results were filtered for clinical trials to eliminate other types of publications (eg, review articles, meta-analyses, case reports) and minimize risk of bias. For all citations obtained, MeSH terms, abstracts, and when necessary the article text, were reviewed. Citations were excluded if they were not English-language or were duplicate PubMed entries; if they reported trials in animals or in healthy subjects without AD; if they included subjects with psoriasis, asthma, or hand eczema; or if TCI, or TCS, were not the active treatment being assessed. Meta-analyses/review articles that did not present new, previously unpublished data were excluded, as well as retrospective studies, case reports, and studies of 10 or fewer patients.

Finally, we excluded studies that did not include safety data, were less than 12 weeks’ duration, or did not include pediatric patients (<12 yrs of age).

### Summary of safety data from TCI and TCS trials

Long-term safety data from the TCI and TCS trials that met inclusion criteria were summarized descriptively; statistical analyses were not performed due to the inconsistency in reporting of these data. We limited the focus to safety data that we judged to be most relevant to long-term AD treatment: discontinuations due to AEs, cutaneous AEs (skin infection, atrophy), and systemic AEs (infection, lymphoma, gastrointestinal [GI] events, respiratory tract infections [RTIs]). We did not present the incidence of minor application site reactions (burning, pruritus) that typically occur early in treatment and resolve, potentially atopic or allergic events (allergies, asthma, conjunctivitis) that are common in patients with AD and are generally not related to treatment, or events with unclear origin that are unlikely to be treatment-related (cough, fever, headache, nasopharyngitis, rhinitis).

## Results

### Published trials included in safety summary

The PubMed queries resulted in 221 TCI citations and 487 TCS citations (Figs. 1 and 2). Of these, 21 TCI trials (27 citations [41, 44–69]) met the inclusion criteria for our safety summary and are presented in order of duration in Tables 2 and 3. All 21 TCI trials were published after 2000, and more than half were published after the Boxed Warning was issued in 2006. Six trials (6 citations [50, 61, 70–73]) met inclusion criteria and are included in the TCS safety summary (Table 4), 2 of which were actually TCI trials with a TCS treatment arm as an active comparator (both trials are also included in the TCI safety summary [50, 61]). One long-term TCS trial in pediatric patients was excluded from our summary due to its retrospective design (*n* = 756) [74]. In this study, patients using TCS of any potency were included in the study, and no systemic safety data were reported. All but 1 of the TCS trials included in our summary were published after 2000, but only 1 was published after 2006.

**Table 2:** Study designs for long-term (≥12 weeks) tacrolimus trials in pediatric patients (<12 years) with AD

Trial	Duration (wk)^a^	Baseline AD severity	Age, mean (range)	TCS Use	N	Treatment^b^
Controlled studies						
Paller 2001 [49]	12	moderate to severe (≥4.5 R&L)	6 yr (2–15)	None	118	**tacrolimus** 0.1 % BID (DB) for flares
					117	**tacrolimus** 0.03 % BID (DB) for flares
					116	**vehicle** BID (DB) for flares
Hofman 2006 [50]	28	moderate to severe (≥4.5 R&L)	~6 yr (2–11)	None	133	tacrolimus 0.03 % BID for 3 wk, then **tacrolimus** QD + vehicle QD (DB) for flares
				-	124	hydrocortisone ointment 1 % BID for head/neck and hydrocortisone butyrate ointment 0.1 % BID for trunk/limbs for 2 wk, then **hydrocortisone 1 %** BID (DB) for flares
				None	50	**no treatment** (patients did not have AD)
Paller 2008 [51] Breneman 2008 [52]	42	moderate to severe (mean EASI = ~11)	7 yr (2–15)	None	68	tacrolimus 0.03 % or alclometasone ointment 0.05 % BID for 4 d (DB), then BID (OL) for 2–16 wk until clearance; then **tacrolimus** 0.03 % QD 3x/wk (DB) and tacrolimus BID for flares
					36	tacrolimus 0.03 % or alclometasone ointment 0.05 % BID for 4 d (DB), then BID (OL) for 2–16 wk until clearance; then **vehicle** QD 3x/wk (DB) and tacrolimus 0.03 % BID for flares
Thaçi 2008 [53] Thaçi 2010 [54]	52	mild to severe (≥3 R&L)	7 yr (2–15)	None	125	tacrolimus 0.03 % BID (OL) for 1–6 wk until clearance, then **tacrolimus** 0.3 % 2x/wk (DB) and tacrolimus 0.03 % BID (OL) for flares
					125	tacrolimus 0.03 % BID (OL) for 1–6 wk until clearance, then **vehicle** 2x/wk (DB) and tacrolimus 0.03 % BID (OL) for flares
Uncontrolled studies						
Kubota 2009 [55]	12	moderate to severe (mean EASI = 13)	7 yr (2–15)	None^c^	31	OL tacrolimus 0.03 % QD + TCS (strong or weak) QD for 2 wk, then tacrolimus BID on weekdays and tacrolimus QD + TCS QD on weekends for 2 wk, then **tacrolimus** BID (no TCS) for 2 wk followed by tacrolimus BID (no TCS) for flares
Tan 2004 [56]	24	mild to severe	8 yr (2–15)	None	82	OL **tacrolimus** 0.1 % BID until 1wk after clearance, then **tacrolimus** 0.1 % BID for flares
Kang 2001 [57]	52	moderate to severe (≥4.5 R&L)	8 yr (2–15)	None	255	OL **tacrolimus** 0.1 % BID for flares
Mandelin 2012 [58]	104	moderate to severe (mean EASI = 11)	15 mo (3–24)	NR^d^	50	OL **tacrolimus** 0.03 % BID for 3 wk and then QD until clearance; thereafter BID for flares
Hanifin 2005 [59]	156 (≤196 wk exposure)	mild to severe (R&L)	(2–15 yr)	NR^e^	391	OL **tacrolimus** 0.1 % BID for flares for 3 yr (after 1 yr tacrolimus in unpublished lead-in study)

*N* = safety population

*BID* indicates twice daily, *d* days, *DB* double-blind, *EASI* eczema area and severity index, *mo* months, *NR* not reported, *OL* open label, *PSGA* physicians static global assessment, *pts* patients, *QD* once daily, *R&L* Rajka and Langeland, *TCS* topical corticosteroids, *wk* week(s), *yr* year(s)

^a^For trials of <12 months: duration in weeks = 4 X total months of study. For trials ≥1 year: duration in weeks = 52 X total years of study

^b^To differentiate the long-term study treatments from any short-term lead-in treatments, the long-term treatments are indicated in bold

^c^TCS use per protocol was permitted during the first 4 weeks and prohibited for the remainder of the study

^d^TCS use was permitted (for up to 2 weeks in any 3 months) to treat flares not controlled by study medication; information on the incidence and duration of TCS use was NR

^e^TCS use was not permitted, however an unspecfied number of patients deviated from protocol and used TCS; these patients were not excluded from study summary

**Table 3:** Study designs for long-term (≥12 weeks) pimecrolimus trials in pediatric patients (<12 years) with AD

Trial	Duration (wk)^a^	Baseline AD severity	Age, mean (range)	TCS Use	N	Treatment^b^
Controlled studies						
Ruer-Mulard 2009 [60]	22	mild to severe (mean EASI = ~10)	7 yr (2–17)	NR^c^	134	pimecrolimus 1 % BID (OL) for ≤6 wk until clearance, then **pimecrolimus BID** (DB) for flares
					134	pimecrolimus 1 % BID (OL) for ≤6 wk until clearance, then **pimecrolimus QD** + vehicle QD (DB) for flares
Siegfried 2006 [44]	24	mild to severe (mean IGA = 3)	59 mo (3–140)	40 % of pts	183	**pimecrolimus** 1 % BID (DB) for flares
			62 mo (3–143)	55 % of pts	92	**vehicle** BID (DB) for flares
Zuberbier 2007 [47] Zuberbier 2008 [48]	24	severe (R&L = 8.3)	~8 yr (2–17)	29 % of days	195	prednicarbate cream 0.25 % OL for 7–21 d, then pimecrolimus 1 % BID (DB) until clearance (≥7 d) and **pimecrolimus** BID for flares
				35 % of days	89	prednicarbate cream 0.25 % OL for 7–21 d, then vehicle BID (DB) until clearance (≥7 d) and **vehicle** BID for flares
Sigurgeirsson 2008 [45]	26	mild to moderate (IGA ≤1)	7 yr (1–17)	41 % of pts	256	**pimecrolimus** 1 % BID (DB) for flares
				72 % of pts	265	**vehicle** BID (DB) for flares
Kapp 2002 [41]	52	mild to severe (mean EASI = ~12)	12 mo (3–23)	36 % of pts	204	**pimecrolimus** 1 % BID (DB) for flares
				65 % of pts	46	**vehicle** BID (DB) for flares
Wahn 2002 [46]	52	mild to severe (mean EASI = ~13)	8 yr (1–17)	43 % of pts	474	**pimecrolimus** 1 % BID (DB) for flares
				68 % of pts	237	**vehicle** BID (DB) for flares
Sigurgeirsson 2015 [61]	260	mild to moderate (IGA = 2–3)	7 mo (3–12)	64 % of pts	1205	**pimecrolimus** 1 % (OL) until clearance, and then **pimecrolimus** as needed for flares^d^
				-	1213	hydrocortisone 1 % or hydrocortisone butyrate 0.1 % (OL) until clearance and then **hydrocortisone** as needed for flaresd
Uncontrolled studies						
Kaufmann 2004 [62] Staab 2005 [63]	20	mild to severe (mean EASI = ~17)	(3–23 mo)	NR^e^	188	pimecrolimus 1 % or vehicle BID (DB) for 2–4 wk until clearance, then **pimecrolimus** 1 % BID (OL) for flares for 12 wks and 4 wks with no treatment
Lübbe 2006 [64]	24	mild to severe	15 yr (<1–81)	53 % of pts	947	**pimecrolimus** 1 % BID (OL) for flares
Simon 2006 [65]	24	mild to severe	21 yr (<1–70)	NR^c^	109	**pimecrolimus** 1 % BID (OL) until clearance, then **pimecrolimus** BID for flares
Whalley 2002 [66] Langley 2008 [67]	26	mild to moderate (IGA = 2–3)	~7 yr (<2–17)	None	233	pimecrolimus 1 % BID (DB) for 6 wks, then **pimecrolimus** BID (OL)
					102	vehicle BID (DB) for 6wks, then **pimecrolimus** BID (OL)
Papp 2005 [68] Papp 2005 [69]	52 (≤104 wk exposure)	mild to severe (mean EASI = 5.8)	28 mo (18–41)	28 % of pts	91	**pimecrolimus** 1 % BID (OL) for flares for 1 yr (following 1 yr of pimecrolimus 1 % BID [DB] or vehicle BID in lead-in study [Kapp et al. 2002 [41]]

*N* safety population

*BID* indicates twice daily, *d* days, *DB* double-blind, *EASI* eczema area and severity index, *IGA* investigator’s global assessment, *mo* months, *NR* not reported, *OL* open label, *pts* patients, *QD* once daily, *R&L* Rajka and Langeland, *TCS* topical corticosteroids, *wk* week(s), *yr* year(s)

^a^For trials of <12 months: duration in weeks = 4 X total months of study. For trials ≥1 year: duration in weeks = 52 X total years of study

^b^To differentiate the long-term study treatments from any short-term lead-in treatments, the long-term treatments are indicated in bold

^c^TCS use was permitted to treat flares not controlled by study medication; information on the incidence and duration of TCS use was not reported (NR)

^d^Pimecrolimus and TCS dosing during acute and maintenance phases was per the study country’s label

^e^It was not stated whether TCS use was permitted

**Table 4:** Study designs for long-term (≥12 weeks) topical corticosteroid trials in pediatric patients (<12 years) with AD

Trial	Duration (wk)^a^	Baseline AD severity	Age, mean (range)	TCS Potency [87]	N	Treatment^b^
Controlled studies						
Thomas 2002 [70]	18	mild to moderate (mean SASSAD = ~8–14)	5 yr (1–15)	low (class 7–6)	104	**hydrocortisone** ointment 1 % BID (DB) for 7 d bursts “when required”
		mild to moderate (mean SASSAD = ~9–16)	6 yr (1–15)	mid (class 5–3)	103	alternating (DB) **betamethasone valerate** ointment 0.1 % BID (3 d) and emollient BID (4 d) for 7 d bursts “when required”
Jorizzo 1995 [71]	25	mild to moderate	5 yr (<1–12)	low (class 7–6)	16	**desonide** ointment 0.05 % BID (SB)
				low (class 7–6)	20	**hydrocortisone** ointment 1 % BID (SB)
Hanifin 2002 [72]	44	moderate to severe (mean R&L = 7)	7 yr (<1–17)	mid (class 5–3)	154	fluticasone propionate cream 0.05 % BID (OL) for ≤4 wks until clearance; then **fluticasone** **propionate** BID (DB) decreased over 20 wk to QD 2x/wk; then **fluticasone propionate** QD 2x/wk (OL) for 20 wk
				-	77	fluticasone propionate cream 0.05 % BID (OL) for ≤4 wks until clearance; then **vehicle** BID (DB) decreased over 20 wk to QD 2x/wk; then **fluticasone propionate** QD 2x/wk (OL) for 20 wk
Meta-analysis						
Kirkup 2003 [73]^c^	16	moderate to severe (mean AD^d^ = ~12^e^)	8 yr (2–14)	mid (class 5–3)	136	hydrocortisone 1 % cream BID for 1–2 wk, then **fluticasone propionate** cream 0.05 % BID (DB) for 2–4 wk and then “as required” for flares
				low or mid (class 7–3)	129	hydrocortisone 1 % cream BID for 1–2 wk, then **hydrocortisone 1 % or hydrocortisone butyrate 0.1 %** BID (DB) for 2–4 wk and then “as required” for flares
TCS as active comparator^f^						
Hofman 2006 [50]	28	moderate to severe (≥4.5 R&L)	~6 yr (2–11)	-	133	tacrolimus 0.03 % BID for 3 wk, then **tacrolimus** QD + vehicle QD (DB) for flares
				low (class 7–6)	124	hydrocortisone ointment 1 % BID for head/neck and hydrocortisone butyrate ointment 0.1 % BID for trunk/limbs for 2 wk, then **hydrocortisone 1 %** BID (DB) for flares
				-	50	**no treatment** (patients did not have AD)
Sigurgeirsson 2015 [61]	260	mild to moderate (IGA = 2–3)	7 mo (3–12)	-	1205	**pimecrolimus** 1 % (OL) until clearance, and then as needed for flares^g^
		mild to moderate (IGA = 2–3)	7 mo (3–12)	low or mid (class 7–3)	1213	**hydrocortisone 1 % or hydrocortisone butyrate 0.1 %**(OL) until clearance, and then as needed for flares^g^

*N* = safety population

*AD* indicates atopic dermatitis, *BID* twice daily, *d* days, *DB* double-blind, *EASI* eczema area and severity index, *IGA* investigator’s global assessment, *mo* months, *NR* not reported, *OL* open label, *pts* patients, *QD* once daily, *R&L* Rajka and Langeland, *SASSAD* Six area, six sign atopic dermatitis, *TCS* topical corticosteroids, *wk* week(s), *yr* year(s)

^a^For trials of <12 months: duration in weeks = 4 X total months of study. For trials ≥1 year: duration in weeks = 52 X total years of study

^b^To differentiate the long-term study treatments from any short-term lead-in treatments, the long-term treatments are indicated in bold

^c^Meta-analysis of 2 previously unpublished studies

^d^AD Score (max 21) = Number of body areas affected (max 12) + Sum of erythema, excoriation, and lichenification scores (each graded 0–3) at target area (max 9)

^e^After ‘run in’

^f^TCS treatment was an active comparator arm in 2 TCI trials: Hofman et al. 2006 (also listed in Table 1) and Sigurgeirsson (also listed in Table 2)

^g^Pimecrolimus and TCS dosing during acute and maintenance phases was per the study country’s label

### Study designs

Treatment varied according to individual study designs, but typically a brief twice-daily regimen to control the initial flare was followed by a maintenance period of intermittent, as-needed treatment for flares (study treatments during the maintenance phase are indicated in bold in Tables 2, 3 and 4).

#### TCI trials

Three tacrolimus trials were double-blind, vehicle-controlled (2 tacrolimus 0.03 % vs vehicle [51–54], 1 tacrolimus 0.1 % vs tacrolimus 0.03 % vs vehicle [49]), 1 was double-blind, active- and no treatment-controlled (tacrolimus 0.03 % vs hydrocortisone vs no treatment [50]), and 5 were uncontrolled, open-label [55–59]. Five pimecrolimus studies were double-blind, vehicle-controlled [41, 44–48], and 1 was double-blind, active-controlled (pimecrolimus BID vs QD [60]). One study was open-label, active-controlled (the Petite Study, pimecrolimus vs low- or mid-potency TCS [61]), and 5 were uncontrolled, open-label trials [62–69].

#### TCS trials

Two TCS studies were double-blind, active-controlled: 1 low-potency TCS (hydrocortisone) vs mid-potency TCS (betamethasone valerate) [70], and 1 low-potency TCS (desonide) vs low-potency TCS (hydrocortisone) [71]. One was an open-label, vehicle-controlled trial of mid-potency TCS (fluticasone) vs vehicle [72], and another was a meta-analysis of 2 previously unpublished, double-blind, active-controlled trials of mid- or low-potency TCS (fluticasone or hydrocortisone) [73].

Two additional studies that met inclusion criteria for TCS safety summary were actually TCI studies in which TCS treatment was an active comparator: one was a double-blind trial of tacrolimus vs low-potency TCS (hydrocortisone) vs no treatment [50], and the other was the Petite Study, an open-label trial of pimecrolimus vs low- or mid-potency TCS (hydrocortisone or hydrocortisone butyrate) [61].

We did not identify any published long-term pediatric trials of high-potency TCS.

#### TCS use in TCI studies

All except 3 of the tacrolimus studies prohibited TCS use; 1 study permitted TCS use per protocol during the first 4 weeks [55], 1 permitted TCS use for flares not controlled by study medication [58], and 1 prohibited TCS use but did not exclude an unspecified number of patients that deviated from study protocol and used TCS [59]. In 10 of the 12 pimecrolimus studies, TCS use was permitted for flares not controlled by study medication; in these studies, 28–72 % of patients in any treatment group reported using a TCS (2 trials did not report the incidence of TCS use).

### Number of patients analyzed and duration of treatment

**Fig. 3:**
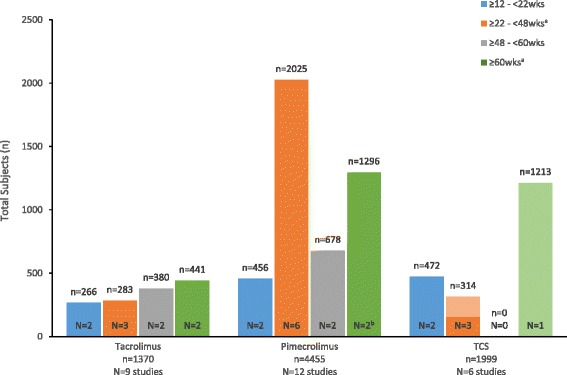
Total subjects included in summary of long-term (≥12 weeks) pediatric trials by therapeutic agent and study duration. ^a^ Lighter shading indicates the proportion of patients that received TCS treatment as an active comparator in studies of a TCI. ^b^ One of the trials was a 1-yr OL extension of a 1-yr DB study. *N* = Number of studies. *n* = Number of subjects in respective treatment group

The TCI studies ranged in duration from 12 weeks to 5 years (260 weeks) and included 5825 pediatric patients with mild to severe AD (tacrolimus studies = 1370, pimecrolimus studies = 4455) (Fig. 3). Six TCS trials (including 2 in which TCS treatment was an active comparator) included 1999 pediatric patients with mild to severe AD. The only TCS study longer than 48 weeks was the Petite study, in which TCS was an active comparator for pimecrolimus [61]; the 1213 TCS patients in this study accounted for more than half of the total number of TCS subjects in the analysis. In contrast, there were 8 TCI trials of >2700 patients with durations of more than 48 weeks [41, 46, 53, 54, 57–59, 61, 68, 69], including a 2-year study (*n* = 91), 4-year study (*n* = 391), and the 5-year Petite study (*n* = 1205).

### Reporting of safety data in the published trials

Reporting of safety data was highly variable from study to study, especially in the TCS studies. While TCI trials usually reported the cutaneous and systemic AEs that occurred most frequently during the study, many TCS studies only reported application-site events, and did not report the incidence of systemic AEs. Further, instead of reporting any AE that occurred frequently, regardless of severity or relation to study treatment, some TCS studies reported only AEs that were classified as serious/severe and/or thought to be treatment-related. Terminology and classification of AEs was inconsistent across all the studies, with some studies reporting incidences of specific AEs, and others reporting incidences of general AE categories (eg, bacterial, viral, or fungal infection). In the studies that reported the incidence of specific AEs, the terminology used for the AEs was not consistent between studies.

### Summary of safety data

Safety data are summarized in Table 5. Studies are listed from shortest to longest duration, and divided by treatment received: tacrolimus (*N* = 1370; 0.03 %, *n* = 524; 0.1 %, *n* = 846), pimecrolimus (*N* = 4455), low-potency TCS (*N* = 400), mid-potency TCS (*N* = 257), low- or mid-potency TCS (*N* = 1342), vehicle + TCS rescue (TCS was permitted to treat flares not controlled by study medication, *N* = 729), vehicle (*N* = 355), and no treatment (*N* = 50; these subjects did not have AD).

**Table 5:**
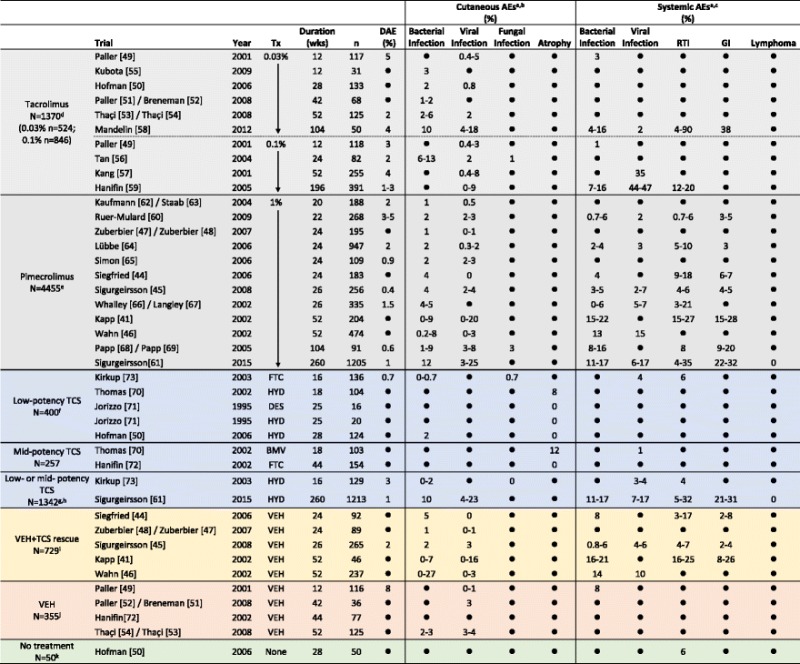
Adverse events reported in long-term (≥12 weeks) pediatric trials of TCI and TCS listed by duration

If trial duration was <1 year and reported in months, the number of weeks was calculated as follows: duration in weeks = 4 X duration in months. If trial duration was ≥1 year, the number of weeks was calculated as follows: duration in weeks = number of years X 52

Only incidences of adverse events (AEs) that were specifically reported to be “0” are indicated as such; AEs that were not a specified study outcome and/or were not reported are indicated as “●”

Incidences of discontinuations due to adverse events (DAEs) and AEs >1 % are rounded to the nearest whole number; incidences are presented as a range when multiple AEs within the same category were reported

^a^ AEs that may have been reported in the studies but are not shown include: application site reactions (burning, pruritus, etc.), potentially atopic or allergic events, or events with unknown/unclear origin (asthma, conjunctivitis, coughing, fever/pyrexia, joint pain, nasal congestion, nasopharyngitis, pharyngitis, rhinitis/coryza, rhinorrhea)

^b^ Cutaneous AE categories

**Bacterial infection** = abscess, bacterial infection, boil, cellulitis, eczema Infected/infection, erysipelas, folliculitis, furuncle, impetigo, infection, pustules, staphylococcal Infection, streptococcal infection, or stye

**Viral infection** = chicken pox, eczema herpeticum, flat warts, herpes simplex, herpes virus infection, herpes zoster, Kaposi’s varicelliform eruption, molluscum, skin papilloma, varicella, viral rash Candida, fungal infection, ringworm

**Atrophy** = antecubital fossae atrophy, atrophy, hypertrichosis, popliteal fossae atrophy, telangiectasia

^c^ Systemic AE categories

**Bacterial infection** = bacterial pneumonia, bronchitis, ear infection, external otitis, infection, laryngitis, sinusitis, Staphylococcus infection, Streptococcus pharyngitis, tonsillitis

**Viral infection** = flu-like symptoms, influenza, influenza-like Illness, viral encephalitis

**Respiratory tract infection (RTI)** = otitis media, respiratory tract infection, upper respiratory tract infection, viral respiratory tract infection

**Gastrointestinal infection (GI)** = diarrhea, gastroenteritis, viral gastroenteritis, vomiting

^d^ Incidences of some viral infections were reported only for the combined tacrolimus group in the Paller study

^e^ Results for the 2 pimecrolimus treatment groups are combined in the Ruer-Mulard and Whalley/Langley studies

^f^ TCS treatment was received In the Hofman study as an active control for tacrolimus

^g^ Patients received either hydrocortisone 1 % (low-potency TCS) or hydrocortisone butyrate 0.1 % (mid-potency TCS)

^h^ TCS treatment was received in the Sigurgeirsson study as an active control for pimecrolimus

^i^ TCS was permitted to treat flares not controlled by study medication

^j^ Tacrolimus was permitted in the Paller/Breneman and Thaçi/Thaçi studies for flares not controlled by study medication

^k^ These subjects did not have AD

*AE* indicates adverse event, *BID* twice daily, *BMV* betamethasone valerate, *DAE* discontinuations due to AE, *DES* desonide, *FTC*, fluticasone propionate, *GI* gastrointestinal, *HB* hydrocortisone butyrate, *HYD* hydrocortisone, *mo* months, *PM* pimecrolimus, *pts* patients, *QD* once daily, *RTI* respiratory tract infection, *TC* tacrolimus, *TCS* topical corticosteroids, *Tx* treatment, *URTI* upper respiratory tract infection, *VEH* vehicle, *wk*, week(s), *yr*, year(s)

To increase clarity and interpretability, AEs were categorized as either cutaneous events (bacterial infection, viral, infection, fungal infection, or atrophy) or systemic events (bacterial infection, viral infection, RTIs, GI events, or lymphoma). A list of the AE terms that were included in each category is provided in the footer of Table 5. When multiple AEs in the same category were reported, incidences are shown as a range. If the incidence of an AE category was not reported in the published study, it could not be assumed to be 0 and is therefore shown as “●”. AE incidences of >1 % were rounded to the nearest whole number.

### Discontinuations due to adverse events

Fourteen of 21 TCI studies reported the incidence of discontinuation due to AEs (DAEs, Table 5); in those studies it was ≤5 %. About half of the DAEs were caused by application-site events (with the exception of 1 tacrolimus study in which all 9 DAEs in the vehicle group were application-site events). In contrast, only 2 TCS trials reported the incidence of DAEs; in those 2 studies, the incidence was <5 %.

### Cutaneous adverse events

The incidence of skin infections in TCI-treated patients was generally low (Table 5), and similar to those reported by vehicle-treated patients. In all but one trial of <1 year duration, ≤5 % of patients reported skin infections (in a 24-week, uncontrolled, open-label tacrolimus study, 13 % of patients reported impetigo [56]). In most longer-term studies (≥1 year duration), skin infection occurred in <10 % of patients. Varicella was reported by 18 % of patients in a 2-year tacrolimus study and 20 and 16 % of pimecrolimus and vehicle patients, respectively in a 1-year study [41, 58]), and impetigo was reported by 27 % of vehicle patients (but just 8 % of pimecrolimus patients) in another 1-year trial [46]. In the 5-year Petite study, rates of skin infection were similar in the pimecrolimus and TCS groups (impetigo was reported in 12 % of pimecrolimus and 10 % of low- or mid-potency TCS patients, and varicella was reported in 25 and 23 %, respectively [61]). There were no reports of skin atrophy in the TCI studies.

Besides the Petite study, the incidence of skin infection in TCS patients was reported in just 2 studies, a 16-week trial and a 28-week trial; the incidence was ≤2 % in those studies. Three TCS studies used various methods to assess skin for signs of atrophy. An 18-week study measured skin thickness with an ultrasound scanner [70] and found that 8 and 12 % of patients using low- and mid-potency TCS, respectively, had >25 % reduction in skin thickness. In a 25-week study of low-potency TCS [71], investigators found no signs of atrophy using an 8x magnifying lamp. In a meta-analysis of 2 16-week studies of low- or mid-potency TCS [72], investigators visually assessed skin and found no evidence of atrophy.

### Systemic adverse events

The most frequently reported systemic AEs in the TCI studies were common childhood infections that were considered unrelated to treatment. Systemic infections were reported in up to 20 % of patients in TCI studies of <1 year duration; higher incidences were reported in some longer studies. In one 1-year study, 27 and 25 % of patients in the pimecrolimus and vehicle groups, respectively, reported an RTI, and 28 and 26 % reported a gastrointestinal (GI) event [41]. Almost half of patients in a 4-year, open-label tacrolimus study reported a viral infection [59], and in a 2-year, open-label tacrolimus study up to 90 % of patients reported an RTI, and 38 % reported a GI event [58].

In the 5-year Petite study, up to 17 % of pimecrolimus and TCS patients reported some type of systemic bacterial infection, and 17 % reported influenza-like illness [61]. Up to 35 and 32 % of pimecrolimus and TCS patients, respectively, reported RTIs, and up to 32 and 31 % had GI events. Besides the Petite study, just 2 TCS studies of 16- and 18-week durations reported incidences of systemic AEs of ≤6 % [70, 73].

### Effects on adrenal and immune system function

The Petite study investigated the effects of up to 5 years of AD treatment on growth rate and immune system function in a large population of infants, and found no difference in growth rate between the pimecrolimus and TCS treatment groups [61]. Similarly, immunologic investigation showed that infants in the pimecrolimus and TCS groups developed antibody titers to common vaccine antigens that were similar to each other and to what would be expected in the general population.

Prior to the Petite study, there were few published studies that evaluated the effects of long-term TCS treatment on immune system or HPA-axis function in pediatric patients. In a 44-week study of mid-potency TCS, 2 patients from a small cohort that were administered a cosyntropin stimulation test (CST) had reduced serum cortisol responses [72]. In a 28-week study, neither tacrolimus nor low-potency TCS interfered with immune response following vaccination [50], and a third study found that long-term (up to 2 years) treatment with pimecrolimus did not interfere with the development of systemic immune responses in young children [68].

### Ocular risk

None of the TCI and TCS trials we identified in our systematic literature search assessed glaucoma or cataracts.

### Lymphoma risk

There were no reports of lymphoma in any of the TCI and TCS trials, including the 5-year Petite Study (Table 5).

## Discussion

Given the increasing frequency of AD, primary care physicians will continue to play an important role in its management. Some clinicians, patients, and/or parents have concerns about the safety of topical AD treatments, especially in children who have an increased risk of percutaneous absorption and systemic exposure. These safety concerns are also increased with long-term daily maintenance therapy.

We conducted a systematic search for safety data from published pediatric TCI and TCS clinical trials of at least 12 weeks duration, and identified 27 clinical trials that met our inclusion criteria. Twenty-one TCI studies included 5825 pediatric patients, >2700 of whom were followed for 48 weeks to 5 years. Six TCS studies were limited to low- to mid-potency products and included 1999 pediatric patients, more than half of whom received TCS as an active comparator in a study designed to evaluate the safety of pimecrolimus. Most TCI studies included a vehicle control group, while all but one TCS study lacked a vehicle control.

Safety data were generally well reported in the TCI studies, including the incidences of cutaneous and systemic infections. Less than 5 % of patients in the TCI studies discontinued due to AEs. Incidences of cutaneous AEs were similar in TCI and vehicle treatment groups, and were within expected range given the predisposition of the AD patient to cutaneous infections [75]. Systemic AEs that occurred were common childhood infections and not considered to be related to treatment; incidences were similar in treatment and vehicle groups. Extracutaneous viral infections were reported in 47 and 90 % of patients in 2 uncontrolled tacrolimus studies; these incidences are not unexpected given that children under age 5 are expected to have 3 to 6 respiratory infections per year [76, 77]. In contrast, safety data in TCS trials were not as well reported: systemic and cutaneous AEs were mostly unreported, and DAE incidence was addressed in just 2 trials. None of the TCS studies assessed the incidence of glaucoma or cataracts.

The inconsistency of safety data reporting in the trials may be due to timing, as more than half of the TCI studies were published after 2006, when the Boxed Warning for TCIs increased the focus on their safety and appropriate use. In contrast, only 2 TCS studies (including the Petite Study) were published in or after 2006. More recent trials tend to have improved protocols with standardized and validated methods for assessing safety data. This is especially true following the establishment by the NIH in 2000 of a clinical trial registry (ClinicalTrials.gov), and FDA-mandated expansions in 2007 of registry requirements, including disclosure of study design. Further, journal criteria for publication continue to become more rigorous, requiring more clear and complete reporting of data as well as clinical trial registration prior to publication.

According to current treatment guidelines, low-to-mid potency TCS should be used as first-line, short-term treatment of flares [3, 8, 78]. Choice of potency depends on the severity, extent, and site of the flare; the least potent TCS that can control the symptoms should be used. No TCS is indicated for >4 consecutive weeks of use, and few are approved for patients younger than 2 years of age because of risk of atrophy and rebound effects. For mild AD, long-term control is often possible with intermittent TCS for flares and trigger avoidance, bleach baths, and daily moisturizers. Moderate-to-severe AD usually requires long-term daily maintenance therapy with intermittent TCS or TCIs, which are most useful as steroid-sparing agents. The regimen and choice of product for maintenance therapy depends on factors including patient preference, access to medications, cost, and a careful evaluation of benefits versus risks.

Our review supports the long-term safety of TCIs and low-to-mid potency TCS in pediatric patients with AD. Long-term treatment with TCIs and intermittent use of low-to-mid potency TCS was generally well tolerated in 27 trials of >5800 and >1900 pediatric patients, respectively, with no evidence of cutaneous atrophy or cumulative systemic exposure and no reports of lymphoma. This reflects preclinical animal studies that detected malignancy signals only after systemic TCI exposure was high enough to cause immune suppression. This exposure was much higher than the negligible systemic exposure detected after twice daily topical administration (the highest blood concentrations reported for infants with pimecrolimus 1 % range from 1.8 to 4.14 mg/ml, and the average maximum concentration with tacrolimus 0.03 % was 3 % of that observed in pediatric liver-transplant patients receiving oral tacrolimus) [17, 79–84].

Many recent meta-analyses and reviews have also contributed to the body of evidence that has filed to detect increased lymphoma risk with TCIs [18–39]. One study compared the incidence of TCI-associated malignancies reported to multiple TCI AE databases and found a rate similar to or lower than the expected rate of malignancy in the general population [17], and another compared incidences of lymphoma in health insurance claims databases and did not find an increased risk among patients treated with TCIs versus TCS [85]. And no increased risk of malignancy was detected in 7457 children enrolled as of May 2014 in the ongoing prospective 10-year observational cohort study of children with a history of AD and pimecrolimus use (Pediatric Eczema Elective Registry, PEER) [86].

A decade’s worth of clinical experience, epidemiological data, postmarketing surveillance, and adverse event database monitoring have failed to demonstrate a causal relationship between TCI use and malignancy, yet TCI labelling continues to include a Boxed Warning. The biggest impact of the warning is to limit patient access to the most well-studied medications for long-term maintenance AD treatment, especially in children.

## Conclusions

This comprehensive literature review supports the long-term safety of TCI and low- to mid-potency TCS therapy in children with AD, with no evidence of cutaneous atrophy or cumulative systemic exposure and no reports of lymphoma. We found comparatively limited data on the long-term safety of mid- to high-potency TCS. Our findings are not reflected by the current TCI labelling and Boxed Warning; therefore we hope our review facilitates the rescindment of the Boxed Warning.

## Abbreviations

AD: atopic dermatitis
AE: adverse event
BID: twice daily
BMV: betamethasone valerate
d: days
DAE: discontinuation due to adverse effect
DB: double-blind
DES: desonide
EASI: eczema area and severity index
FTC: fluticasone propionate
GI: gastrointestinal
HB: hydrocortisone butyrate
HYD: hydrocortisone
IGA: investigator’s global assessment
Mo: months
NR: not reported
OL: open-label
PM: pimecrolimus
PSGA: physician’s static global assessment
Pts: patients
QD: once daily
R&L: Rajka and Langeland
RTI: respiratory tract infection
SASSAD: six area, six sign atopic dermatitis
TC: tacrolimus
TCI: topical calcineurin inhibitor
TCS: topical corticosteroids
Tx: treatment
URTI: upper respiratory tract infection
VEH: vehicle
Wk: week(s)
Yr: year(s)
